# Are patient falls in the hospital associated with lunar cycles? A retrospective observational study

**DOI:** 10.1186/1472-6955-4-5

**Published:** 2005-10-17

**Authors:** René Schwendimann, Franco Joos, Sabina De Geest, Koen Milisen

**Affiliations:** 1 Institute of Nursing Science, University of Basel, Bernoullistrasse 28, 4056 Basel, Switzerland; 2 Stadtspital Waid Zurich, Switzerland; 3 Institute of Astronomy, Swiss Federal Institute of Technology, Zurich, Switzerland; 4 Center for Health Services and Nursing Research, Catholic University of Leuven, Leuven, Belgium

## Abstract

**Background:**

Falls and associated negative outcomes in hospitalized patients are of significant concerns. The etiology of hospital inpatient falls is multifactorial, including both intrinsic and extrinsic factors. Anecdotes from clinical practice exist in which health care professionals express the idea that the number of patient falls increases during times of full moon. The aim of this study was to examine in-hospital patient fall rates and their associations with days of the week, months, seasons and lunar cycles.

**Methods:**

3,842 fall incident reports of adult in-patients who fell while hospitalized in a 300-bed urban public hospital in Zurich, Switzerland were included. Adjusted fall rates per 1'000 patient days were compared with days of the week, months, and 62 complete lunar cycles from 1999 to 2003.

**Results:**

The fall rate per 1000 patient days fluctuated slightly over the entire observation time, ranging from 8.4 falls to 9.7 falls per month (P = 0.757), and from 8.3 falls on Mondays to 9.3 falls on Saturdays (P = 0.587). The fall rate per 1000 patient days within the lunar days ranged from 7.2 falls on lunar day 17 to 10.6 falls on lunar day 20 (P = 0.575).

**Conclusion:**

The inpatient fall rates in this hospital were neither associated with days of the week, months, or seasons nor with lunar cycles such as full moon or new moon. Preventive strategies should be focused on patients' modifiable fall risk factors and the provision of organizational conditions which support a safe hospital environment.

## Background

Falls occur frequently in hospitalized patients. Patient fall rates in hospital settings vary from 2.2 to 9.1 falls per 1000 patient days depending on patient populations and disease groups [1-7]. The etiology of falls in hospitalized patients is multifactorial consisting of both intrinsic and extrinsic risk factors [8-10]. Studies on hospital falls that focus on occurrences over time are limited to the frequencies of falls during the hours of the day [1,5-7,11,12], and to specific time spans e.g. number of falls within the first week of hospitalization [2,4,7,13].

Reasons for the fluctuation in fall-rates over time have been debated, but never scientifically researched. There exist anecdotes from health care professionals in our clinical practice that express the idea that the number of patient falls increasing during times of full moon. One survey indicated that specifically mental health professionals including psychologists, nurses and others held the personal belief that lunar phases affect patient's behavior [14]. However, only one study could be found which reports an increased frequency in patient accidents in a hospital, of which 90% were patient falls, during times of full moon and new moon [15].

Associations between lunar cycles and health conditions, however, such as increased phone call rates by females to a crisis-call centre, higher frequency in misbehaviors in institutionalized patients, greater behavioral deterioration in patients with schizophrenia, increased occurrence in gout attacks, and higher frequencies in the number of appointment requests in thyroid outpatients; rates of gastrointestinal bleeding; multiparae delivery rates; and numbers of births, have been reported [16-23].

Several beliefs, theories and hypotheses regarding lunar impact on the human body have been generated throughout the history of human kind. Assumptions such as the "Gravitational pull hypothesis" or the "Tidal force hypothesis" were extensively analyzed but their impact on the human organism could not be empirically substantiated [24]. A series of studies have rejected the hypothesis of a lunar influence on human health in view of the following: suicide rates [25,26]; violent behavior and aggression [27,28]; agitation in nursing home residents [29]; use of psychiatric community services [30]; psychiatric hospital admissions [31]; frequency of admissions toemergency care [32]; volume of patients admitted to emergency departments [33]; cardiopulmonary arrests in emergency departments [34]; incidence of myocardial infarction and sudden cardiac death [35]; onset of spontaneous pneumothorax [36]; survival time for breast cancer patients [37]; number of surgical complications [38]; postoperative nausea and vomiting [39]; workload on labor and delivery wards [40]; and number of deliveries [41].

There is evidence stating that professionals believe there are correlations between falls and times of the full moon, although an association between patient falls during hospitalization and lunar cycles, especially the influence of the full moon, has not yet been scientifically explored. We hypothesized that no relationship exists between falls in hospitalized patients and lunar cycles. The aim of this study was therefore to examine in-hospital patient fall rates and their associations with days of the week, months, seasons and lunar cycles.

## Methods

### Study sample and setting

We conducted a retrospective analysis of all registered in-patient falls amongst the adult patients hospitalized on the general internal medicine, surgery and geriatric rehabilitation wards of a 300-bed public hospital, which provides medical services for the inhabitants of the Northern part of the city of Zurich, Switzerland. The observation period was from January 1, 1999 to December 31, 2003. Ethical approval was granted by the Ethics Committee of the City hospitals of the City of Zurich.

### Variables and measurements

Patient falls were defined as "an incident in which a patient suddenly and involuntary came to rest upon the ground or surface" and were registered regularly by the nurses discovering the fall. We retrieved the number of registered patient falls occurring during hospital stay from the incident report data system of the quality management department, and screened administrative patient data to determine daily number of hospitalized patients, individual length of patient stay, and daily bed occupancy rates. We identified the dates of the synodic lunar months within the study period, based on the European Southern Observatory Munich Image Data Analysis System (ESO-MIDAS). One synodic lunar month counts 29.53 days (29 d. 12 h. 44 m.) which is the period of time required for the moon to travel from one position relative to the sun as seen from the Earth (e.g. full moon) and return to the same position. The day counts started with the new moon at day 0 until the full moon between day 14 and 15 and ended before the next new moon on day 28 or day 29.

### Data analysis

We calculated fall rates per 1000 patient days to adjust for number of falls per day and number of hospitalized patients per day. To examine the pattern of fall rates over time, we calculated mean (including standard deviations (SD), and 95% confidence intervals (CI)) fall rates per day of the week, month and season throughout the study period. To model the rate of falls per 1000 patient days with lunar days, days of the week, and months as predictor variables, we used a general linear model. Statistical tests and confidence intervals were calculated two sided, and p-values <0.05 were considered statistically significant. All analyses were performed using SPSS (12.0).

## Results

The 5 year study period included 1,826 observation days. During this time a total of 34,970 patients were hospitalized (mean age: 67.3 (SD 19.3) years, female: 53.6%), accounting for 431,149 patient days. Mean length of stay was 12.3 (SD 14.4) days. The hospital bed occupancy rate was 86.2% (Median: 86.6%). Overall, a total of 3,843 falls were registered, affecting 2,512 (7.2%) patients.

### Number of hospitalized patients

The number of hospitalized patients per day ranged over the entire study period from 182 to 279 with a mean of 236 patients (SD 17, median 237). The mean number of hospitalized patients per day of the week varied significantly from 221 (SD 14) on Sundays to 244 (SD 16) on Thursdays (p < 0.001). The mean number of hospitalized patients per month varied significantly from 220 (SD 17) per day in August to 247 (SD 16) per day in February (p < 0.001).

### Incidence of patient falls over time

**Figure 1:**
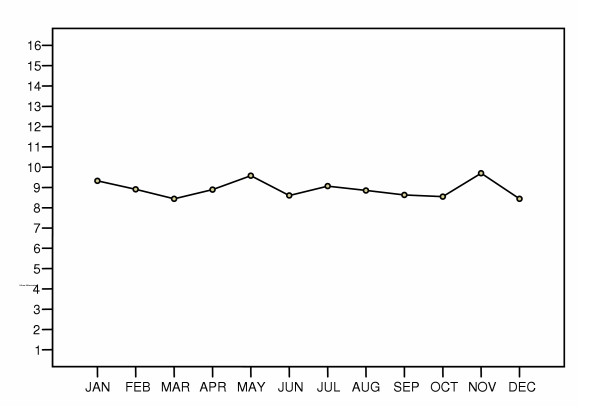
Mean fall rates per month (1999–2003).

Throughout the study period, the frequency of daily falls ranged from zero to eight falls. The overall mean fall rate was 8.9 (SD 6.4) falls per 1000 patient days. Per day of the week, the mean fall rate ranged from 8.3 (SD 6.9) falls per 1000 patient days on Mondays to 9.3 (SD 6.7) falls per 1000 patient days on Saturdays (df 6; F = .778; p = .587). Per month, the mean fall rate ranged from a low of 8.4 (SD 6.1) falls per 1000 patient days in December to a high of 9.7 (SD 6.8) falls per 1000 patient days in November (df 11; F = .682; p = .757) (Fig. 1). The mean fall rate per 1000 patient days per season of the year varied although not significantly: The lowest rate was in Autumn, with 8.7 (SD 6.2) falls/1000 patient days; In Winter there were 9.0 (SD 6.2) falls; the highest rate of falls was in Spring with 9.1 (SD 6.8), and in Summer there were 9.0 (SD 6.2) (df = 3: F = 0.213; p = 0.887).

### Falls, lunar cycle, and variation in time

**Figure 2:**
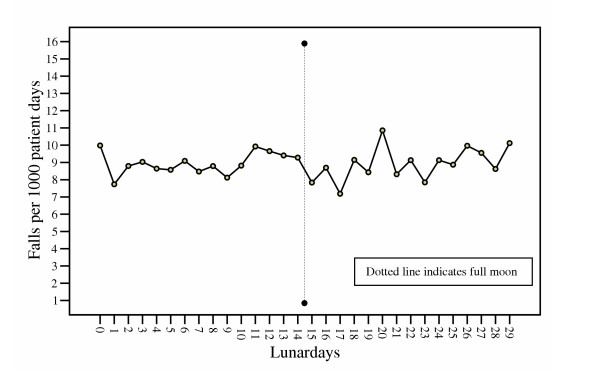
Mean fall rates per lunar day (1999–2003).

Sixty two complete synodic lunar cycles were observed during the study period. The first full moon was observed on January 2, 1999 (first new moon: January 17, 1999) and the last full moon was seen on December 8, 2003 (last new moon: December 23, 2003). Within the days of the lunar cycle, the variation in mean fall rates per 1000 patient days was not significant. The lowest rate was 7.2 (SD 6.0) falls on lunar day 17, and the highest rate was 10.6 (SD 6.3) falls on lunar day 20 (df 29; F = .929; p = 0.575) (Fig. 2).

The fall rates per 1000 patient days, lunar days, and variation in time including days of the week, and months of the year, showed neither a statistically significant main effect, nor a statistically significant interaction between the variables under study (Table 1).

**Table 1:** Associations between falls/1000 patient days, lunar cycle, days of the week & month

	**df**	**F-value**	**P-value**
Corrected model^a)^	1503	0.989	0.560
Lunar day	29	0.973	0.509
Days of the week	6	0.545	0.773
Month	11	0.368	0.967
Day of the week & month	66	1.040	0.403
Lunar day & days of the week	174	1.077	0.283
Lunar day & month	318	1.046	0.345
Lunar day, days of the week & month	899	0.949	0.721

^a) ^R^2 ^= 0.822 (adjusted R^2 ^= -.010)

## Discussion

Throughout the 5 year study period, no significant association was found in the incidence rate of hospital in-patient falls occurring during the time period of the full or new moon, neither was periodicity demonstrated for days of the week, months or seasons of the year. Despite significant fluctuations of the hospital's patient occupancy per day of the week and month, the patient fall rates remained relatively stable during the entire study period.

Our results contrast with the one other study that addressed the relationship between patient falls and lunar cycles [15]. Sutton et al reported significant findings in view of increased accident rates during the seven days prior to a full moon and the seven days prior to the new moon. In contrast, we examined whether there were associations between fall rates per day during the lunar cycle, throughout 62 lunar cycles.

In general, our findings are concordant with all other studies that, as with our study, did not show an association between lunar days and patient related events such as hospital admissions, emergency department visits, accessing psychiatric services, and violent behavior [28,30-33].

We assume that the belief of some health care professionals that frequency of in-hospital fall accidents increases with the time of the full moon rely on non-specific, non systematic observations within the realm of everyday practice. Such beliefs are probably influenced by lay press reports that highlight bizarre unusual activities when the moon is full [42]. Empirical evidence shows that the etiology of falls during hospitalization is multifactorial. Clinically identifiable risk factors such as impaired mobility, impaired mental status, special toileting needs, psychotropic medications, and a past history of falling have been consistently found to be relevant for predicting future falls [8,10,43]. Of note is that it has recently been shown that hospital system related factors such as nurse staffing and nurse skill mix also influence the frequency of patient falls [44-46].

The challenge for healthcare professionals will be to support patient safety and quality of care by early identification of patients at risk for falling, and implement interventions to prevent falls and related injuries.

## Conclusion

The in-patient fall rates were neither associated with days of the week, months, or seasons, nor with lunar cycles such as the full moon or new moon. Preventive strategies should be focused on assessment of patients' modifiable fall risk factors, and the provision of organizational conditions which support a safe hospital environment.

## Competing interests

The author(s) declare that they have no competing interests.

## Authors' contributions

RS contributed to the conception, design, data collection, analysis, interpretation of data, and drafted the manuscript. FJ contributed to the data collection and analysis. SDG contributed to the design, interpretation of data, and critical revision of the manuscript. KM contributed to the analysis, interpretation of data, and manuscript preparation. All authors gave final approval for this version of the manuscript to be published.

## Pre-publication history

The pre-publication history for this paper can be accessed here:


